# Supplementary material for: Neuronal parts list and wiring diagram for a visual system
Source: Nature. 2024 Oct 2;634(8032):166–80. doi: 10.1038/s41586-024-07981-1 (PMC11446827; doi:10.1038/s41586-024-07981-1)

*L*4

predicate precision: 96%, recall: 89%

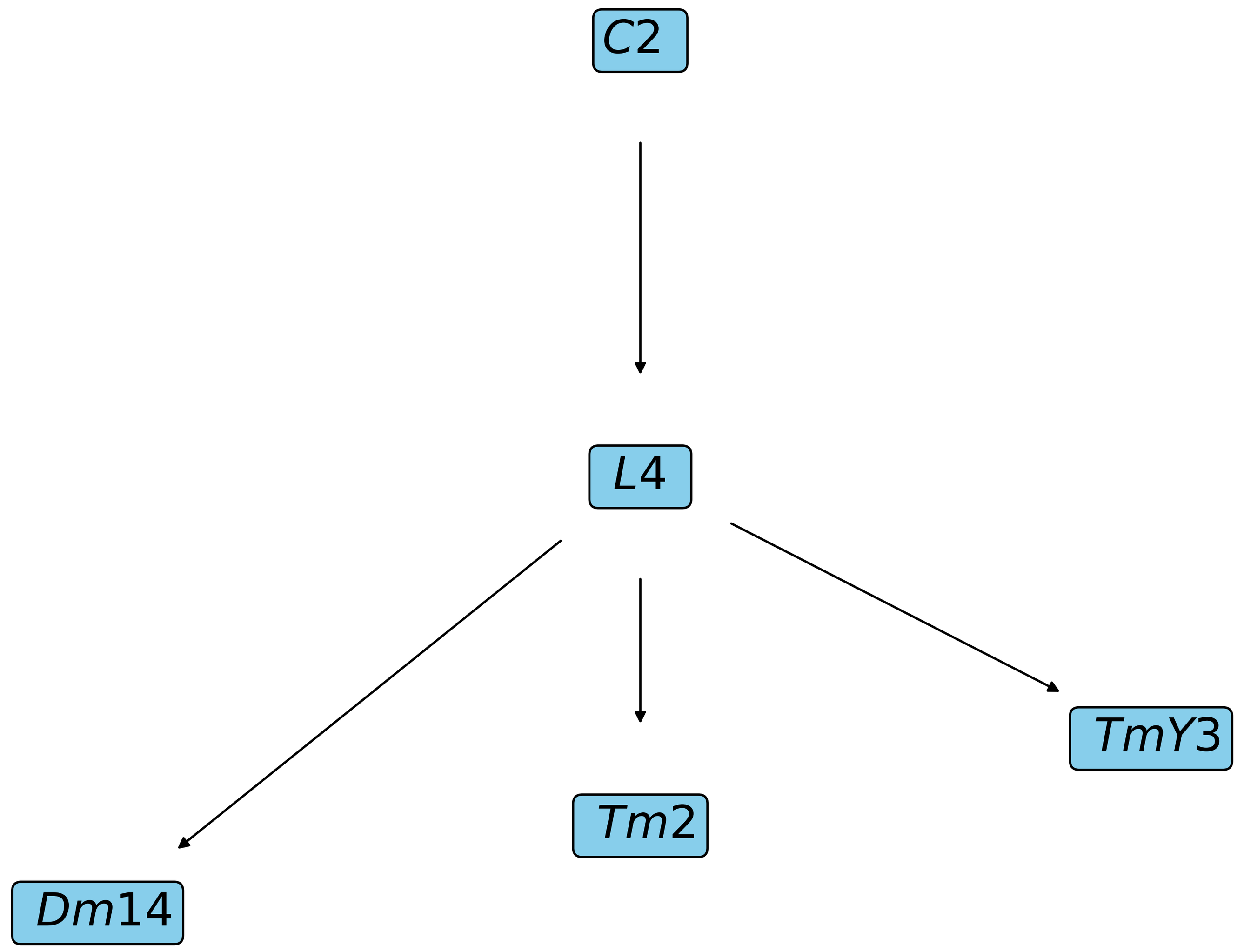

avg. stats

cells	724
NT prediction	ACH (78%)
top in/out regions	ME (90%) / ME (86%)
in/out degree	6 / 25
in/out synapses	20 / 104
cable len	270μ
surface	894μ^2
volume	95μ^3

morphology

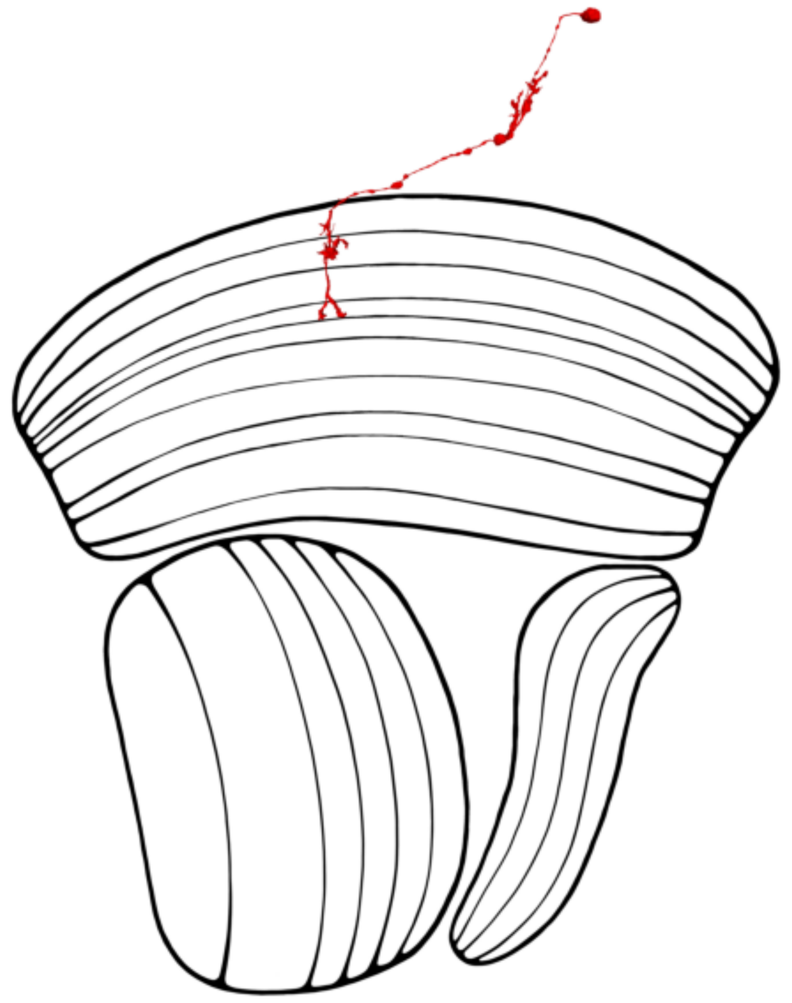

anterior view

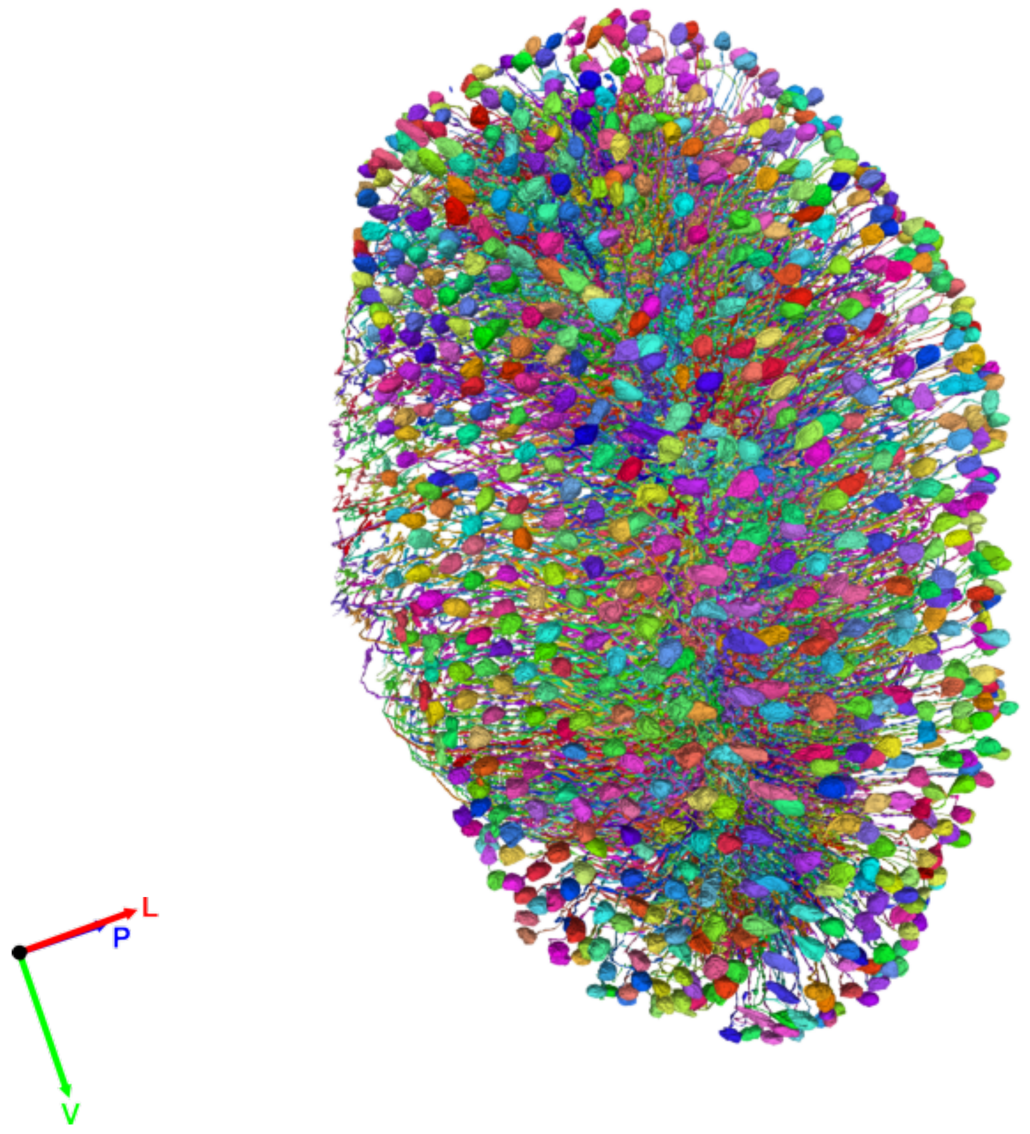

ventral view

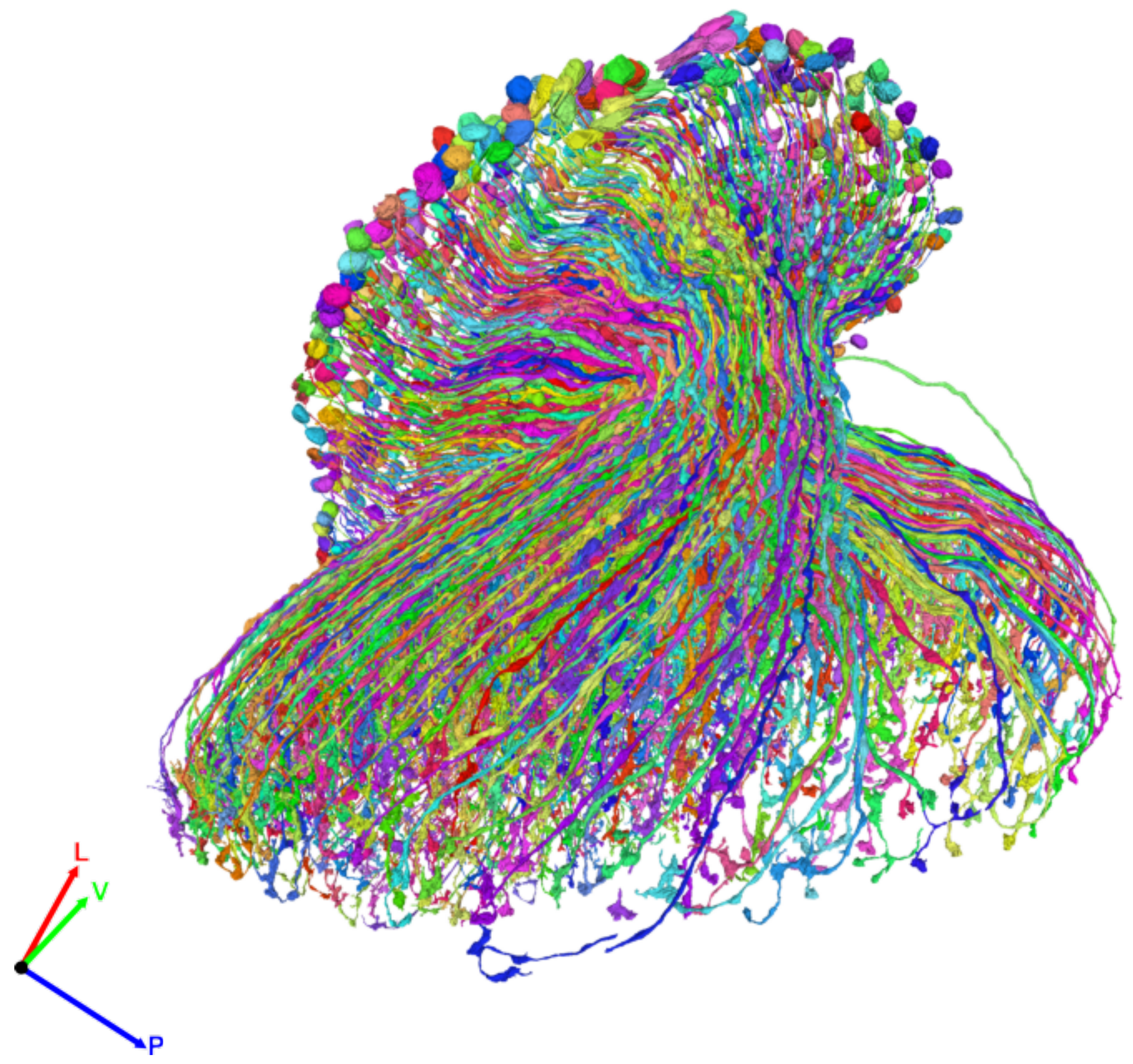

side view

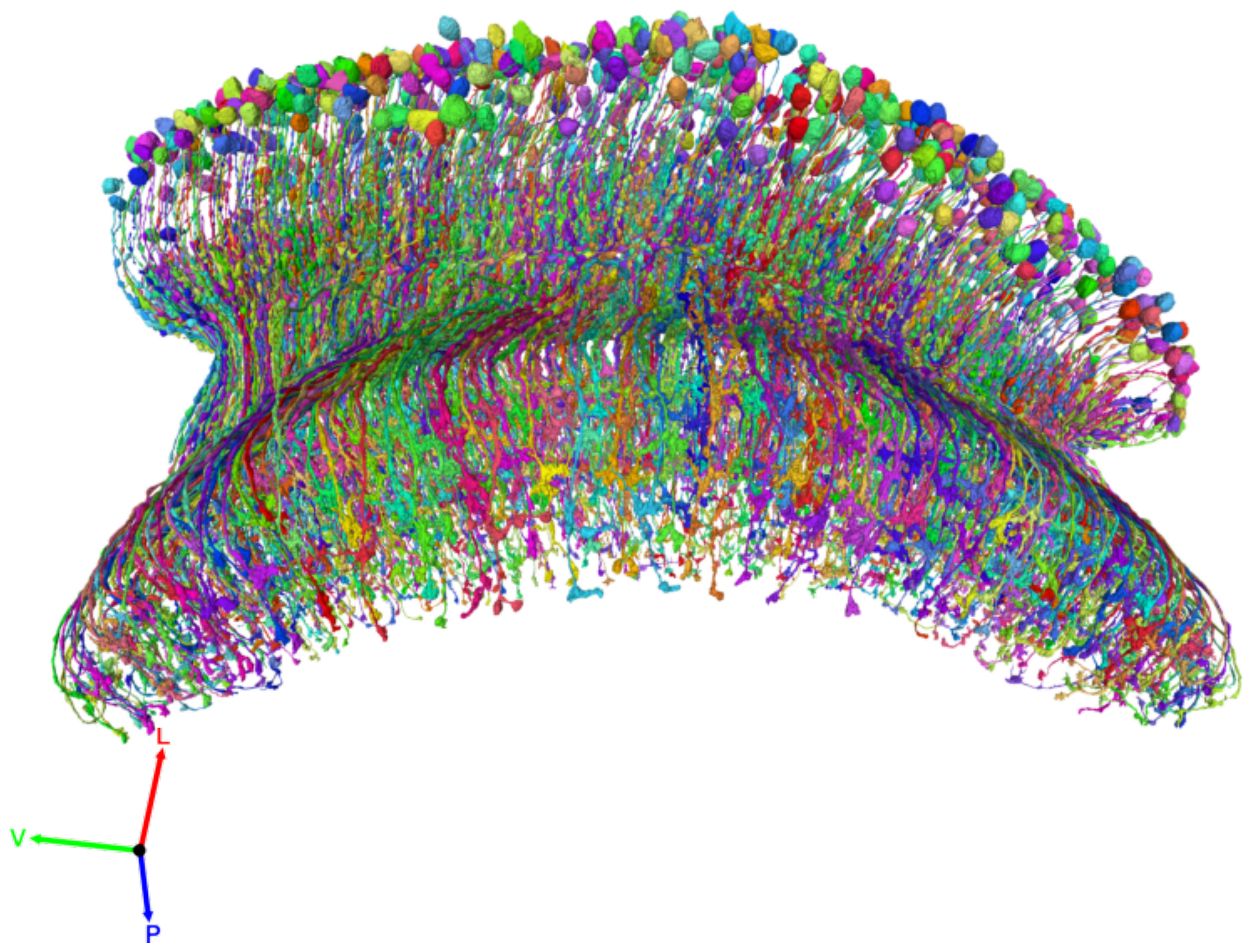

Supplement: Supplementary file 5 — Cards for all cell types. Each card has 6 panels (clockwise): logical connectivity predicate diagram, numeric stats, illustration of a typical cell, followed by 3D rendering of all cells from three orthogonal viewpoints. Also available at Codex. [file 41586_2024_7981_MOESM5_ESM.zip › pdf/L4.pdf]
